# Quantification of lean and fat tissue repletion following critical illness: a case report

**DOI:** 10.1186/cc6929

**Published:** 2008-06-17

**Authors:** Clare L Reid, Peter R Murgatroyd, Antony Wright, David K Menon

**Affiliations:** 1 Division of Anaesthesia, University of Cambridge, Box 93, Addenbrooke's Hospital, Hills Road, Cambridge CB2 0QQ, UK; 2 Wellcome Trust Clinical Research Facility, Box 127, Addenbrooke's Hospital, Hills Road, Cambridge CB2 0QQ, UK; 3 MRC Human Nutrition Research, Elsie Widdowson Laboratory, Fulbourn Road, Cambridge CB1 9NL, UK

## Abstract

**Introduction:**

Muscle wasting is a recognised feature of critical illness and has obvious implications for patient rehabilitation and recovery. Whilst many clinicians believe lean tissue repletion to be a slow process following critical illness, and a probable explanation for poor functional recovery of patients many months after resolution of the illness, we have found no studies quantifying body composition changes during patient recovery.

**Methods:**

A combination of assessment techniques were used to monitor changes in body composition (that is, fat, water, protein and mineral), following intensive care unit (ICU) discharge, in a 38-year-old female recovering from extrapontine myelinolysis. Assessments were made at discharge from the ICU and then again 1 month, 3 months, 6 months and 12 months later. Functional recovery (respiratory muscle and hand-grip strength) and quality of life (36-item Short-form Health Survey) were assessed at these same timepoints.

**Results:**

Twelve months after discharge from the ICU, and despite an extensive rehabilitation programme and improvements in respiratory muscle and hand-grip muscle strength, our patient was unable to return to full-time employment and continued to complain of fatigue. She had successfully regained weight and was back to her pre-illness body weight. Body composition measurements showed that an incredible 73% of the weight gained was due to an increase in body fat.

**Conclusion:**

It is difficult to extrapolate the results of a single case to the wider ICU population, not least because the present patient sustained a significant neurological injury, but our data are the first to support the long-held belief that patient weight gain following critical illness is largely attributable to a gain in fat mass. The magnitude of body composition changes in the present patient are startling and support the need for longitudinal body composition data in a wider ICU population.

## Introduction

Functional and psychological recovery can be delayed following critical illness [1-4]. Underfeeding is common in critical illness [5-7] and patients lose a significant amount a weight during their intensive care unit (ICU) stay [8] – a large proportion of which is attributable to the depletion of lean tissue, particularly skeletal muscle mass [9-11]. Such weight loss might provide a plausible explanation for the functional impairment seen in post-ICU patients, but there is evidence that patients usually regain the weight they had lost over their acute illness [12]. Clinicians generally accept, however, that while patients regain weight during recovery they do not replenish lean tissue mass. To the best of our knowledge no studies have documented temporal changes in body composition following discharge from the ICU.

We quantified muscle wasting, using a novel ultrasound technique [13], in a patient with extrapontine myelinolysis admitted to our critical care unit for mechanical ventilation. Following discharge from the ICU, the patient's body composition was assessed at 1 month, whilst still an inpatient, and at 3, 6 and 12 months following discharge home. Functional recovery (respiratory muscle [14] and hand-grip strength [15]) and quality of life (36-item Short-form Health Survey [16,17]) were assessed at these same timepoints. Despite the neurological nature of this case, the patient was expected to make a full neurological recovery within the 12-month follow-up period. A case report of extrapontine myelinolysis, similar in severity to that of our patient, documented complete recovery at 6-week follow-up [18].

## Materials and methods

### Patient details

A 38-year-old female presented with a 2-week history of neurological symptoms (unsteady gait, dizziness, slurred speech, vertigo and vomiting) and severe hyponatraemia (102 mmol/l). She was found to have Addison's disease and was commenced on replacement therapy. Despite correcting the sodium according to national guidelines (for example, 1.0 mmol/l/hour to a maximum of 12 mmol/24 hours) her neurological symptoms worsened and the patient required admission to the ICU. Magnetic resonance imaging analysis showed florid basal ganglia signal changes consistent with extrapontine myelinolysis [19]. The patient remained on the ICU for 33 days, during which time she developed sepsis and methicillin-resistant *Staphylococcus aureus *pneumonia. She required mechanical ventilation for 13 days. The patient remained in hospital for 75 days after leaving the ICU but following an intensive rehabilitation programme was discharged to her own home, independent in activities of daily life.

During her ICU stay the patient received hydrocortisone and fludrocortisone (1 g intravenously twice daily and 50 μg orally once daily, respectively) in line with a diagnosis of Addison's disease. At discharge to the ward, the corticosteroid prescription was amended (hydrocortisone, 20 mg orally three times daily; fludrocortisone, 50 μg orally once daily). Prior to discharge home and throughout the 12-month follow-up period, the patient was maintained on hydrocortisone (10 mg, 5 mg, 5 mg orally three times daily) and fludrocortisone (100 μg orally once daily).

### Ultrasound measurement of muscle wasting

Ultrasound was used to monitor muscle wasting throughout the ICU stay. Measurements were made daily for the first 5 days, and then every 1 to 3 days thereafter. Three ultrasound measurements of muscle depth were performed over the anterior surface of the biceps (mid-upper arm), forearm and thigh, according to the technique previously described [13]. The mean values from the three sites were then combined and the results expressed as the percentage change of the initial total muscle thickness.

### Body composition

A combination of body composition techniques was used to determine the body fat and the fat-free mass. These techniques are widely used to assess changes in body composition in various populations but none have been validated in post-ICU patients, particularly during the early days and weeks following discharge when the patients' hydration status may adversely influence measurements. At each visit, dual-energy X-ray absorptiometry (DXA) and air displacement plethysmography were performed. Total body water was measured using a stable isotope dilution technique [20].

### Air displacement plethysmography

The body density was assessed with an air displacement plethysmograph (BodPod; Life Measurement Instruments, Concord, CA, USA). The BodPod was calibrated prior to each procedure. The patient entered the chamber wearing a swimming suit and swim cap, and two body-volume assessments were made. Siri's two-compartment formula was used to calculate the percentage body fat from the body density [21]. From the percentage body fat and the body weight, the total fat mass (kg) and the total fat-free mass (kg) were calculated.

### Total body water

Body water was measured using a stable isotope dilution procedure. The patient received an oral dose of deuterium oxide (0.07 g/kg body weight) and saliva samples were collected at baseline (predose) and at 4, 5 and 6 hours after the dose. The concentration of deuterium in each sample was measured using isotope ratio mass spectrometry as described elsewhere [20], and the pool size was calculated. The hydration fraction of the fat-free mass was assumed to be 0.73, and the fat mass was calculated as the difference between the fat-free mass and the body weight.

### Dual-energy X-ray absorptiometry

A whole-body DXA scan was performed using a GE Lunar Prodigy (GE Medical Systems, Madison, WI, USA) and was analysed using software version 8.1 to estimate the bone mineral mass, the bone mineral content, the fat mass and the fat-free mass. The DXA device measures the attenuation of the two energy X-ray beams crossing the tissue. This measurement allows partitioning between bone versus soft tissue and fat versus lean tissue in pixels of the body where there is no overlaying calcified tissue.

The assessment techniques described above are frequently combined as part of a four-compartment model that is often considered the gold standard in body composition [22]. For the purpose of this case, however, we present the absolute fat mass and fat-free mass data derived from DXA. Since this technique has not been validated in our patient group, the BodPod and total body water measurements were used to test the reproducibility of the measurements. Concordance correlation coefficients [23] – specifically the precision – between methods were excellent (DXA versus BodPod, 0.999; BodPod versus total body water, 0.990; and total body water versus DXA, 0.993).

### Functional recovery

The maximal inspiratory pressure [14] was measured using a Morgan Pmax Monitor (PK Morgan, Kent, UK) to provide an objective measure of respiratory muscle strength. Hand-grip strength was measured on a portable electronic hand-held dynamometer (Department of Medical Physics, Queen's Medical Centre, Nottingham, UK) according to a methodology previously described [15].

### Quality of Life

The 36-item Short-form Health Survey was used to quantify physical and mental well-being during the follow-up period [16,17]. The 36-item Short-form Health Survey is a self-administered questionnaire that comprises eight dimensions: physical functioning, social functioning, role limitations due to physical problems, role limitations due to emotional problems, general mental health, energy and vitality, bodily pain, and general health perceptions. The questionnaire is scaled from 0% (poor health) to 100% (good health) using an algorithm.

### Ethics

The present study was approved by the Cambridgeshire 2 Research Ethics Committee. Informed consent was obtained from the next of kin for the patient's inclusion in the ICU phase of the study. Once the patient regained capacity, written informed consent was obtained directly from her.

## Results

**Figure 1:**
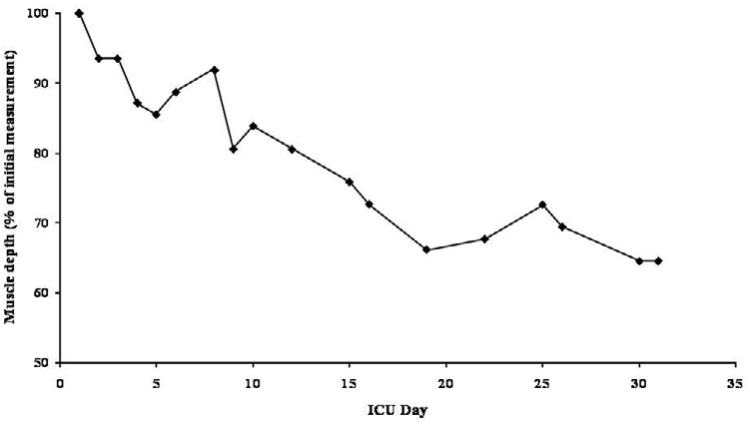
Changes in skeletal muscle depth. Change as a percentage of the initial measurement over the course of the intensive care unit (ICU) stay.

In keeping with previous studies in critically ill patients, the present patient lost a significant amount of weight and lean tissue. On admission her weight was 69.0 kg (body mass index, 25.3). During her 33-day stay on the ICU the patient lost 11.2 kg total weight (16.2% weight loss) or, perhaps more importantly, 36% of her peripheral skeletal muscle mass (Figure 1). Following discharge to the ward, the patient commenced an intensive rehabilitation programme and an energy-dense (40 kcal/kg), high-protein (1.5 g/kg) nutritional support regimen to meet increased nutritional requirements and to facilitate weight gain.

**Figure 2:**
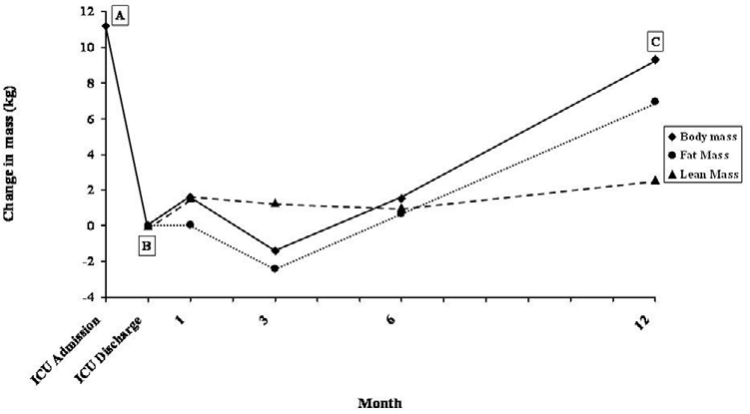
Changes in body mass and composition. Change in mass relative to the time of intensive care unit (ICU) discharge. A, pre-illness weight, 69 kg; B, weight at discharge from the intensive care unit, 58.3 kg; C, weight at 12 months after ICU discharge, 67.1 kg.

Changes in body mass and composition relative to the time of ICU discharge are shown in Figure 2. At 12 months the patient had successfully gained weight (67.1 kg; body mass index, 24.8) and was within 2 kg of her pre-illness weight. Figure 2 clearly illustrates that the total weight gain was closely paralleled by a gain in fat mass. The whole-body lean tissue increased by 2.5 kg over the 12-month period. Between ICU discharge and 1 month (prior to commencing rehabilitation), the lean tissue increased 1.57 kg. There was a subsequent fall in lean tissue mass (-0.55 kg) between 1 month and 6 months, despite an intensive physical rehabilitation programme and a nutrient-dense nutritional support regimen. The lean tissue mass increased a further 1.53 kg at 12 months.

**Figure 3:**
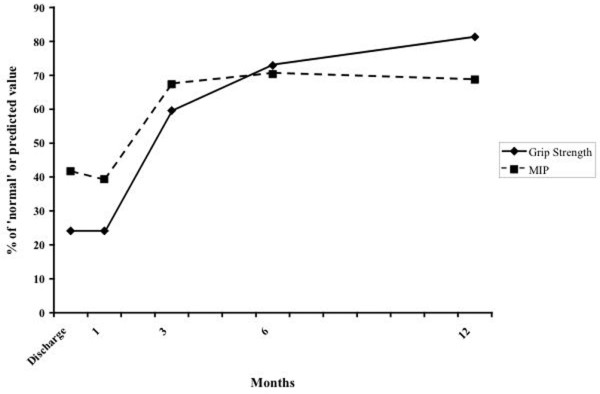
Changes in functional recovery during the 12-month follow up. Change in respiratory muscle and hand-grip strength. MIP, maximal inspiratory pressure.

Despite the lack of lean tissue repletion, the patient demonstrated signficant improvements in both respiratory muscle and hand-grip strength (Figure 3). The maximal inspiratory pressure increased from 41.7% to 70.3% of the predicted value during the follow-up period, while the patient's hand-grip strength increased from 24% to 81.3% of the predicted value (Figure 3).

**Figure 4:**
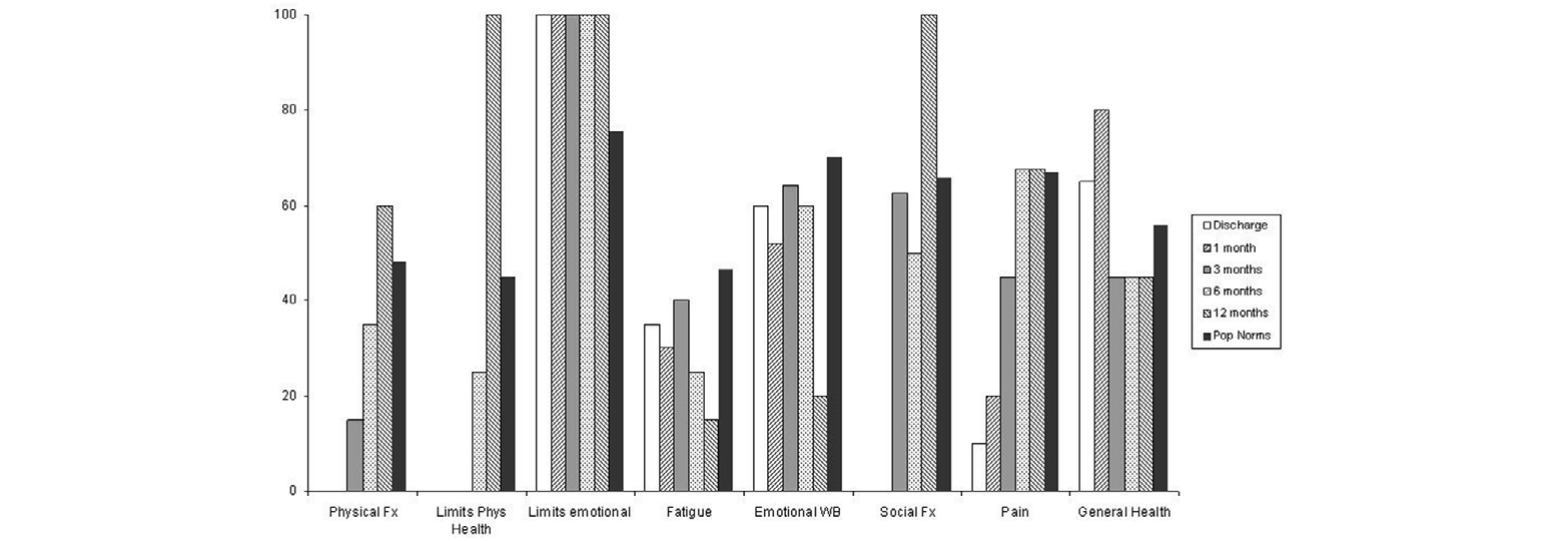
Changes in the quality of life. Changes reported using the 36-item Short-form Health Survey. Physical fx, physical functioning; Limits phys health, role limitations due to physical problems; Limits emotional, role limitations due to emotional problems; Fatigue, energy and vitality; Emotional WB, emotional well-being/mental health; Social fx, social functioning; Pop norms, population norms [24].

In contrast, the 36-item Short-form Health Survey failed to show improvements in all areas (Figure 4). When the eight survey dimensions were examined individually, improvements were seen in physical functioning, social functioning, role limitations due to physical problems, and bodily pain. The patient perceived a worsening, however, of her mental health, energy and vitality, and general health during the 12-month follow-up. Since the 36-item Short-form Health Survey has been used previously to assess ICU patient recovery, population norms from a group of patients following severe sepsis [24] have been included for comparison. At 12 months the present patient was independent in all activities of daily living but was unable to return to full-time work as an office administrator due to ongoing problems with fatigue.

## Discussion

The nutritional support provided to acutely sick patients in the ICU frequently fails to meet their nutritional needs [7,25,26]. Patient nutritional status consequently worsens during the ICU stay. Malnutrition in these patients has been shown to negatively impact short-term clinical outcomes, including the risk of complications, ICU and hospital lengths of stay and mortality [27,28]. Weight loss >10 kg has been reported [8] but it is the dramatic loss of lean tissue within this total weight loss that undoubtedly has the greatest implications for patient recovery and rehabilitation [29,30]. Total body protein losses of up to 16% have been reported; 67% of this loss was from skeletal muscle [30].

Morbidity, mortality, functional capabilities and quality of life following critical illness have been reported previously, with studies consistently showing that recovery is frequently protracted, taking up to 2 years, particularly in patients who experience a prolonged ICU length of stay [1,2,8,31]. There are a small number of studies that show patients can successfully regain weight during the recovery period [12,32], but, surprisingly, no studies have explored changes in body composition, particularly lean tissue repletion, and the possible relationship with clinical outcome following critical illness.

Using a combination of body composition assessment techniques, we have quantified perhaps what many in this field had suspected – lean tissue repletion during rehabilitation and recovery from critical illness is minimal. Although critically ill patients are a complex patient group, this observation is in line with semi-starvation/refeeding studies conducted in healthy subjects during the 1950s [33]. This Minnesota study demonstrated that when fat repletion was at 100%, lean tissue recovery was <40% [34]. The timescale for tissue repletion was only 12 weeks in these healthy, fully mobile subjects, however, compared with the 12-month follow-up period in the present study.

Subsequent work in healthy subjects has shown that the pattern of fat and lean tissue depletion and repletion is determined by an individual's initial body composition, specifically their percentage body fat, independent of their calorie supplementation [35-37]. While the metabolic response to critical illness is very different from that seen in simple starvation, we have shown previously that body composition does influence the rates of muscle wasting during an ICU stay – leaner patients have significantly greater rates of wasting [11]. Exploring whether the same holds true for tissue repletion will be difficult to determine, not least because pre-illness body composition data rarely exist for many ICU patients. Even though we had anticipated a disparity between fat and lean tissue repletion in the present patient, we were still surprised by the magnitude of body composition changes we observed – although the results of a single case must be interpreted with caution. Of the total weight gained, only 27% (2.5 kg) represented lean tissue.

A further consideration when viewing the body composition data for the present patient is the long-term corticosteroid therapy she received. In the acute setting, hydrocortisone therapy has been associated with increased muscle protein catabolism [38] – although the rates of muscle wasting seen in the present patient during her ICU stay were consistent with those reported previously [11]. There are no studies documenting body composition changes in patients with Addison's disease receiving long-term replacement therapy. Hydrocortisone therapy, however, has been associated with muscle wasting, weight gain and alterations in adipose tissue metabolism and distribution [39-41]. The possible influence of corticosteroid therapy on body composition changes seen in our patient therefore cannot be ignored.

While the simple measures of respiratory muscle and hand-grip strength improved over time, they do not provide an accurate measure of fatigue. Clearly the patient was able to undertake activities of short duration, as reflected in her ability to be independent in activities of daily living, including bathing, dressing and feeding for example [42]. More prolonged activities or those requiring greater physical exertion, however, were still beyond the capabilities of this patient. The quality of life data for this patient is largely in keeping with what has been shown during the recovery of other ICU populations [24,31]. Interestingly, however, the patient showed improvements in the functional assessments yet her perception was of a worsening in energy and vitality over the follow-up period. This is consistent with the poor lean tissue repletion during this time. Whilst we might wish to assume an association between the lack of lean tissue repletion and the ongoing fatigue reported by the present patient, we cannot exclude the influence of psychological factors – or indeed any central neurological deficits remaining from the extrapontine myelinolysis.

## Conclusion

To the best of our knowledge we are the first to quantify body composition changes following critical illness. As a result of these findings we are currently undertaking a study to examine body composition changes in a larger cohort of critically ill patients. In addition to the rapid measures of muscle strength used here we shall also use assessments of longer duration (that is, 6-minute walk test), which will provide a better measure of fatigue. Finally, we hope to monitor a subset of patients for at least 2 years to establish when, or even if, patients replenish lean tissue lost during critical illness.

## Key messages

• Weight gain following critical illness is largely attributable to an increase in the fat mass.

• Poor lean tissue repletion and quality of life, and functional impairment persist at 12 months following critical illness.

• Further longitudinal body composition studies are required in the post-ICU patient.

## Abbreviations

DXA = dual-energy x-ray absorptiometry; ICU = intensive care unit.

## Competing interests

The authors declare that they have no competing interests.

## Authors' contributions

CLR conceived of the study, participated in its design and coordination, and drafted the manuscript. PRM carried out the DXA and air displacement plethysmography assessments, analysed the body composition data and helped to draft the manuscript. AW carried out the total body water assessments, analysed the body composition data and helped to draft the manuscript. DKM participated in the study design and helped to draft the manuscript. All authors read and approved the final manuscript.
